# Good communication and trust relationships with women are critical for HIV positive pregnant woman

**DOI:** 10.18332/ejm/146167

**Published:** 2022-10-07

**Authors:** Georgia Pontiki, Katerina Lykeridou, Victoria G. Vivilaki

**Affiliations:** 1 Department of Midwifery, School of Health and Care Sciences, University of West Attica, Athens, Greece; 2 Midwifery Department, Elena Venizelou Maternity Hospital, Athens, Greece

**Keywords:** HIV positive women, pregnancy, midwifery, communication skills, antiretrovial therapy

HIV is undoubtedly a pressing issue of womens health as women represent 47% of new HIV cases^1^. Antiretroviral therapy (ART) has altered HIV contamination to a manageable chronic health problem, on the contrary, the possibility of transmission to the unborn child is 15–45% via gestation, childbirth, or breastfeeding^2^, when not confronted early on. On a global scale, the number of new HIV cases in children has decreased to 47%^3^, due to the increased availability of ART to pregnant women. Compliance with ART therapy is probably the main factor that ensures the best results of the therapeutic regimens^4^.

Communication between health professionals and HIV positive pregnant women is fundamental and essential for their compliance to ART. Therefore, trust relationships built between HIV positive pregnant women and health professionals are of great gravity^5^. Health professionals should demonstrate understanding and empathy and encourage them to express their fears and worries about the regimen and its impact on their health and that of their children. Furthermore, it is of great importance to respect these women and to recognize the difficulties they are going through. The set-up of support programs for HIV positive pregnant women in the hospitals is also essential, and helps their compliance to ART^6^.

The midwives who provide service to women are a trustworthy source of information and advice and, therefore, need to be actively involved in pharmacovigilance and pharmacoepidemiology networks, to receive comprehensive training in ART, and to be empowered to take responsibility for ensuring the generation of high-quality data in terms of ART during pregnancy and lactation. By building trust, they can also be an important source of care and supportive consultation for HIV/AIDS^7^. The acquirement of specialized knowledge of the disease by the midwives is of great importance, as is the empowerment of positive attitude to the programs of vertical transmission decrease^8^. Positive enhancement, empathy, and recognition of the special needs of the HIV-positive pregnant women by the midwives result in the continuation of care^9^. Furthermore, they also result in the enhancement of the communication and the support by the husband/partner and the family at the time of diagnosis and search of care^10^.

The holistic approach of HIV-positive pregnant women before and after childbirth is the fundamental point of guarantee for their compliance to ART. Therefore, health professionals should develop relationships that are based on mutual trust and respect to their needs and recognize in time the need to support people who must comply with the suggested ART regimen^11^. The cooperation between health professionals and the pregnant women and the ‘significant others’ of the supportive environment is required in order to decrease barriers when possible and enhance compliance to the therapy.

Compliance to ART is a complex behaviour, that is determined by factors that are related to the patient, the health professionals, and the health systems^12-14^. The role of health professionals and especially midwives towards compliance to the ART, is essential important for building trust and guaranteeing the smooth management of the disease.

## Data Availability

Data sharing is not applicable to this article as no new data were created.

## References

[1] World Health Organization (2021). HIV/AIDS: Key facts.

[2] Pontiki G, Sarantaki Α, Lykeridou Α (2021). Συμμόρφωση στην Αντιρετροϊκή Αγωγή: Εμπόδια κατά την Εγκυμοσύνη. Hellenic Journal Of Nursing.

[3] Irshad U, Mahdy H, Tonismae T (2021). HIV In Pregnancy.

[4] World Health Organization (2021). World health statistics 2021: monitoring health for the SDGs, sustainable development goals.

[5] Lan YL, Yan YH (2017). The Impact of Trust, Interaction, and Empathy in Doctor-Patient Relationship on Patient Satisfaction. Journal of Nursing and Health Studies.

[6] Igwegbe AO, Ugboaja JO, Nwajiaku LA (2010). Prevalence and determinants of non-adherence to antiretroviral therapy among HIV-positive pregnant women in Nnewi, Nigeria. International Journal of Medicine and Medical Sciences.

[7] World Health Organization (2013). Counselling for Maternal and Newborn Health Care: A Handbook for Building Skills.

[8] Meilani N, Setiyawati N, Barasa, Onyapidi S (2019). Midwife’s Role in the Mother-to-Child Transmission Prevention Program in Primary Health Care in Yogyakarta. Jurnal Kesehatan Masyarakat Nasional (National Public Health Journal).

[9] Kelly C, Alderdice F, Lohan M, Spence D (2013). 'Every pregnant woman needs a midwife'--the experiences of HIV affected women in maternity care. Midwifery.

[10] Jeremiah RD, Patel DR, Chirwa E (2021). A randomized group antenatal care pilot showed increased partner communication and partner HIV testing during pregnancy in Malawi and Tanzania. BMC Pregnancy Childbirth.

[11] British HIV Association (2020). British HIV Association guidelines for the management of HIV in pregnancy and postpartum 2018 (2020 third interim update).

[12] Kleinsinger F (2018). The Unmet Challenge of Medication Nonadherence. Perm J.

[13] de los Rios P, Okoli C, Young B (2020). Treatment aspirations and attitudes towards innovative medications among people living with HIV in 25 countries. Popul Med.

[14] Clark L, Karki C, Noone J (2020). Quantifying people living with HIV who would benefit from an alternative to daily oral therapy: Perspectives from HIV physicians and people living with HIV. Popul Med.

